# Gender Determination Through Dental Morphology: A Cross-Sectional Study

**DOI:** 10.7759/cureus.71073

**Published:** 2024-10-08

**Authors:** Mohammad Qali, Samaa Alsaraf, Hashem Alsaegh

**Affiliations:** 1 Department of Surgical Sciences, College of Dentistry, Health Sciences Center, Kuwait University, Kuwait, KWT; 2 Dentisrty, Ministry of Health, Kuwait, KWT

**Keywords:** anatomy, dental, gender, morphology, teeth

## Abstract

Objectives: Microesthetics portrays the tooth morphology, shape, size, shade, and color. The debate of whether microesthetics may or may not be related to gender is the main focus or interest of our study. The aim of our study is to assess if dentists are able to identify the gender of the patient by evaluating the dental morphology using intraoral photographs only (retracted lips and cheeks).

Materials and methods: This study was a retrospective cross-sectional survey among general dentists and dental specialists practicing in Kuwait. A sample of 151 participants was approached through a multi-stage random sampling method. Data were collected through personal visits to each of the public dental centers. Of these 151 selected, a sample of 10 participants was randomly selected to repeat their participation and answer the questionnaire a second time 2 months after their initial partaking. The statistical analysis for the study was conducted using chi-square tests of goodness of fit and Pearson chi-square. The objectives of this were to compare the dentists' current performance to the task to see if it was due to chance, to compare their previous performance to chance, and to compare their previous and current performances using the McNemar test to determine if any significant differences existed.

Results: In our study, tooth morphology was linked to gender significantly by the participants, which showed that despite some differences, it may still be identified. However, orthodontists did not perform significantly better than other specialties.

Conclusion: In conclusion, our study showed that dentists are able to identify or determine gender based on photographs, which is inconsistent with our starting hypothesis. Overall, the study's findings suggest that both orthodontists and dentists from other specialties could identify an individual's gender based on smiling photographs.

## Introduction

The tooth form plays a crucial role in the harmony of the smile and the face as a whole [1]. When planning a treatment, dentists must consider societal perceptions of aesthetics, harmony, balance, and proportion [2,3]. Therefore, smile components are considered a strong contribution to achieving an attractive face and pleasant smile. This is corroborated by the macroesthetic, miniesthetic, and microesthetic categorizations of the Aesthetic Dentofacial Analysis, by Dr. David Sarver. Microesthetics portrays the tooth morphology, shape, size, shade, and color [4]. The debate of whether microesthetics may or may not be related to gender is the main focus or interest of our study.

Some have proposed that the shapes of teeth should reflect the patient's emotional characteristics, aligning with the "theory of temperament," which classifies temperaments into categories such as sanguine (dynamic), choleric (strong), melancholic (sensitive), and phlegmatic (peaceful) [5,6]. Also, another theory that was correlated to gender, which is a theory that was dependent on a stereotype, proposed that women had more rounded, tapering, ovoid teeth, whereas men presented with square angular teeth. And so, many dental schools teach students to consider gender when selecting tooth molds [6,7]. Women are perceived as more likely to have smaller teeth. However, numerous papers have cited that, despite the various theories, such correlations might not exist in nature [8-11]. In addition to that, different studies have shown that a patient's gender cannot be distinguished by looking at their teeth [1,12].

The aim of our study is to assess if dentists are able to identify the gender of the patient by evaluating the dental morphology using intraoral photographs only (retracted lips and cheeks). The hypothesis of our study is that dentists cannot point out the gender of the patient by looking at the tooth/dental morphology exclusively.

## Materials and methods

Study design and selection of participants

This study is a retrospective cross-sectional survey among general dentists and dental specialists practicing in Kuwait. A sample of 151 participants who are working at Kuwait University and 10 government sites, including specialty centers and polyclinics of the Ministry of Health, were approached through a multi-stage random sampling method. Data were collected through personal visits to each of the public dental centers for a period of two months, January and February 2024. Of these selected 151, a sample of 10 participants was randomly selected to repeat their participation and answer the questionnaire a second time 2 months after their initial partaking. The objectives of this were to compare the dentists' current performance to the task to see if it was due to chance, to compare their previous performance to chance, and to compare their previous and current performances using the McNemar test to determine if any significant differences existed.

Sample size considerations

In 2021, there were approximately 1871 dentists working in the government's public health sector, according to the latest manpower statistics of Kuwait (Ministry of Health, 2021). We estimated that sampling about 10% of this population would allow us to generalize our findings to the entire population of dentists working for the Ministry of Health in Kuwait. The random selection of polyclinics was performed using a computer-based random number generator. We also included faculty professors from Kuwait University's College of Dentistry.

Data collection

The period of data collection lasted 4 months, from January to April 2024. Two of the authors distributed the survey around different regions targeting dentists from different specialties. Earlier permission from the chief dentist at each clinic/center was obtained, and the above-mentioned authors checked the completion of each questionnaire. The average time required to complete the questionnaire was 5 minutes, but due to the high number of patients in some clinics, some participants required a longer duration of time.

Ethical considerations

Ethical consent was obtained from the Ministry of Health as well as the Kuwait University College of Dentistry's Research Ethical Review Committee (ethical approval number 493). The ethics form provides information regarding the type of research, the population being studied, and any special considerations (e.g., if any invasive procedures will be implemented in the research). Approval for access to Ministry of Health facilities was also granted by the Ministry of Health ethics committee. The only item of ethical relevance, information that may personally identify the dentist, was excluded from the questionnaire.

Survey instrument

A two-page questionnaire of 10 photographs was designed to allow dentists to evaluate presented cases or photographs. Cases used in this study were treated with orthodontics by the principal investigator to ensure that malocclusion would not introduce bias or affect the participant's answer in determining the gender of the patient. The order in which the cases were presented is randomized. Participants/dentists were asked about their wish to participate in this study and mention their specialty (General Dental Practitioner, Orthodontist, Periodontist, Prosthodontist, Endodontist, Advanced General Dentist, Oral Maxillofacial Surgeon, Pedodontist, Oral Maxillofacial Radiologist, Residents of the Kuwaiti Board of Orthodontics and Dentofacial Orthopedics). The 10 photographs were intraoral photographs showing the teeth and gingiva solely (retracted cheeks and lips to avoid gender identification by looking at the perioral region), where participants had to predict the gender of the patient based on the dental morphology.

Statistical analysis

The statistical analysis for the study was conducted using two primary methods: chi-square tests of goodness of fit and Pearson chi-square. The chi-square goodness of fit tests were applied to assess the within-group ability of dentists from various specialties to identify gender from photographs. Pearson's chi-square test was used for between-group comparisons to determine if there were significant differences in identification abilities across the different dental specialties. The McNemar test was used to compare the dentists' performance between the first and second time points for the 10 dentists who repeated their contribution.

## Results

The results were analyzed separately for orthodontists and dentists from all other specialties combined, as shown in Table 1. For the orthodontist's group, (n= 283) 54.4% of the responses correctly identified the gender based on tooth morphology photographs, while (n= 237) 45.6% were incorrect. The within-group p-value of 0.044 suggests that the orthodontists' ability to identify gender from tooth morphology was statistically significant. 

In the group comprising all other dental specialties, (n=533) 53.8% of the responses were correct, and (n= 457) 46.2% were incorrect. The within-group p-value of 0.016 indicates that dentists from other specialties could also identify gender from tooth morphology photographs with statistical significance, although the level of significance was slightly stronger than for the orthodontists (Table 1).

**Table 1:** Dentists' ability to identify patients’ gender based on photographs of tooth morphology A p-value of >0.05 is considered non-significant. N/A: not applicable.

	Were dentists able to identify the gender of the patient based on photographs of tooth morphology shown				Within group p-value	Between group p-value	
	No		Yes				
	N	N%	N	N%				
Orthodontists	237	45.6%	283	54.4%	0.044	0.828	N/A	
All other specialties	457	46.2%	533	53.8%	0.016		N/A	

**Figure 1:**
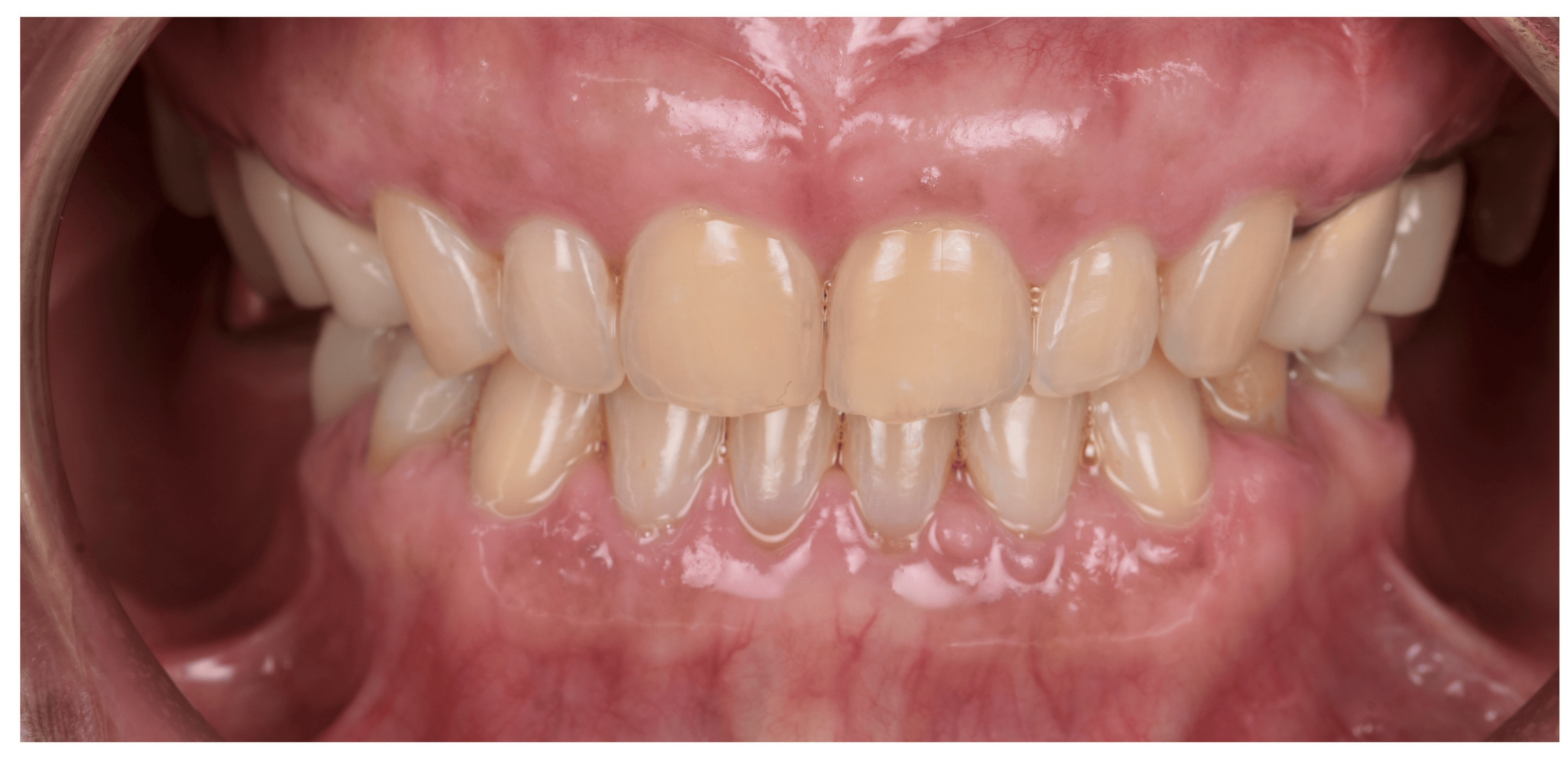
Intraoral photograph (retracted lips): Most cases were identified CORRECTLY as female (Case 2–Table 2)

**Figure 2:**
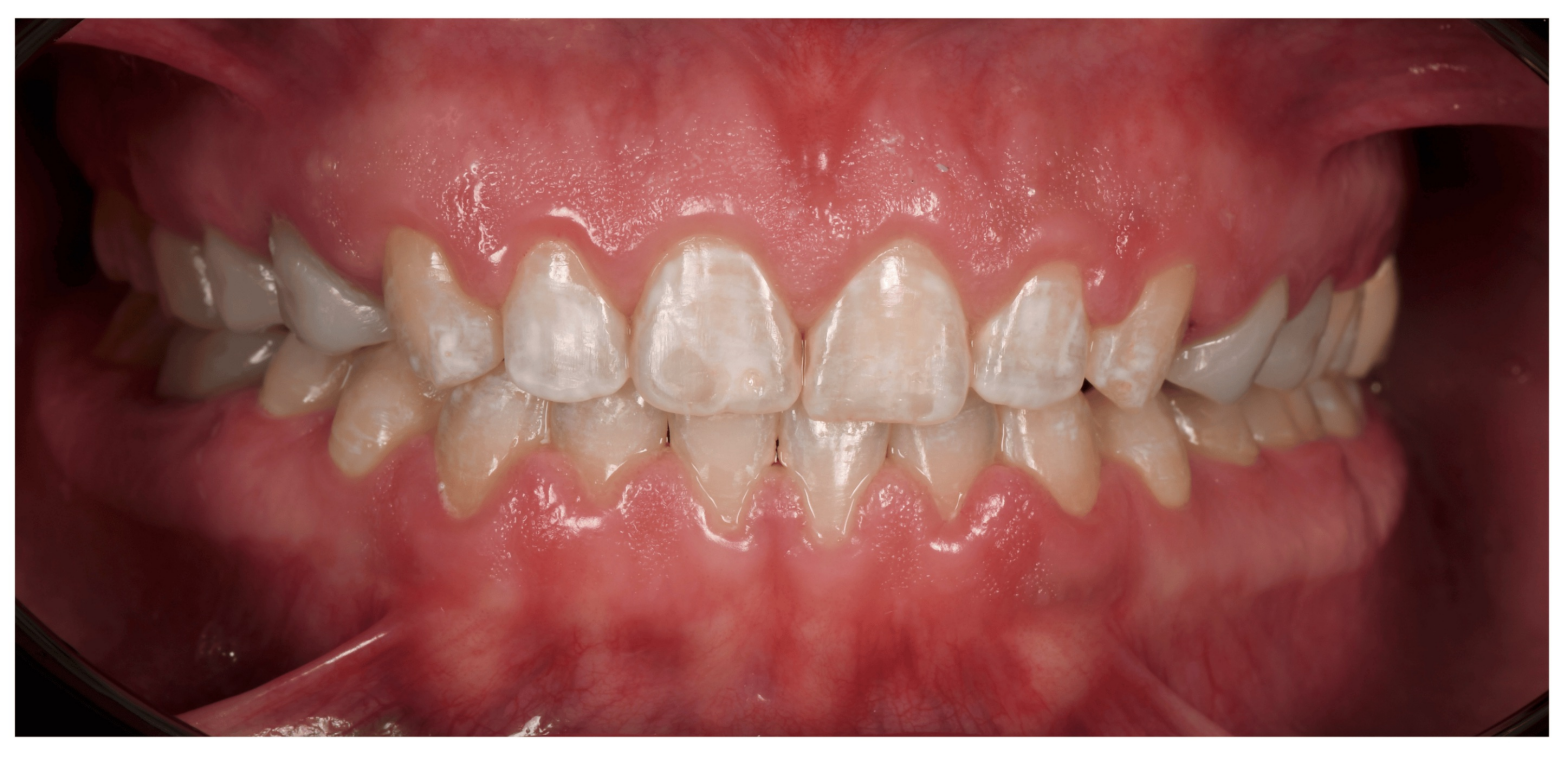
Intraoral photograph (retracted lips): Most cases labeled INCORRECTLY as male (Case 8–Table 2)

However, when comparing the two groups, the between-group p-value of 0.828 revealed no statistically significant difference in their performance. This implies that orthodontists and dentists from other specialties performed similarly in identifying gender based on tooth morphology photographs. Table 2 shows the number of dentists who were able to identify each case correctly. Figure 1 shows the cases that were labeled correctly by dentists most of the time, and Figure 2 presents the cases that were labeled incorrectly (Table 2, Figures 1, 2).

**Table 2:** The number (percentage) of dentists that were able to point out the gender correctly

Case no.	Were all dentists able to identify gender based on the picture shown?	
	No	Yes
	N(N%)	N(N%)
1	66 (43.7%)	85 (56.2%)
2	35 (23.1%)	116 (76.8%)
3	86 (56.9%)	65 (43.0%)
4	49 (32.4%)	102 (67.5%)
5	45 (29.8%)	106 (70.1%)
6	39 (25.8%)	112 (74.1%)
7	102 (67.5%)	49 (32.4%)
8	105 (69.5%)	46 (30.4%)
9	63 (41.7%)	88 (58.2%)
10	104 (68.8%)	47 (31.1%)

Table 3 presents the results of 10 dentists who were asked to identify for the second time the gender of the patient, based on photographs. This task was performed at two different time points.

**Table 3:** Dentists’ performance in identifying patients’ genders from photographs

		N	N%
Were the dentists able to identify gender of the patient the 2^nd^ time	No	53	53.0%
	Yes	47	47.0%
Were the dentists able to identify gender of the patient the 1st time	No	52	52.0%
	Yes	48	48.0%

In the first instance of attempting to identify the patient’s gender, out of 100 answers (10 dentists x 10 cases/photographs), 52 answers (52.0%) were labeled incorrectly, while 48 answers (48.0%) successfully identified the patient’s gender. During the second attempt, 53 answers (53.0%) were incorrect, whereas 47 inputs (47.0%) correctly marked the patient’s gender.

The dentists were nearly evenly split in their ability to identify the patient's gender correctly. However, there was a slight increase in the number and percentage of dentists who could not identify the gender during the second attempt compared to the first (Table 3).

Table 4 presents the results of a binomial test examining whether dentists were able to accurately identify the gender of patients the second time. The test compared the observed proportions to an expected proportion of 0.5 (50%).

**Table 4:** Dentists' performance in identifying patients’ gender: Second Attempt A p-value of >0.05 is considered non-significant. N/A: not applicable.

	Category	N	Observed Prop.	Test Prop.	p-value
Were the dentists able to identify gender of the patient the 2^nd^ time	No	53	0.53	0.50	0.617
	Yes	47	0.47	N/A	N/A

In the second attempt to identify the patient's gender from pictures, dentists were unable to do so correctly for 53 out of the 100 pictures (53%). For the remaining 47 pictures (47%), the dentists successfully identified the gender accurately. The exact two-tailed significance value of 0.617 suggests that the observed proportions of pictures where gender was identified correctly (47%) or incorrectly (53%) did not differ significantly from an expected 50-50 split. In other words, the dentists' performance in identifying gender from these pictures was no better or worse than random chance (Table 4).

During the first attempt with a different set of 100 pictures, dentists failed to correctly identify the patient's gender in 52 pictures (52%). They were successful in accurately identifying the gender for the remaining 48 pictures (48%). Similar to the second attempt, the observed proportions did not deviate significantly from the expected 50-50 split, as indicated by the non-significant p-value.

The dentists faced challenges in accurately identifying the gender of patients and performing at a level consistent with random chance during both attempts. There was no significant improvement or decline in their ability to identify gender between the first and second attempts.

Table 5 presents the results of a statistical test examining whether dentists were able to accurately identify the gender of patients from a set of 10 pictures during the first attempt. For 52 out of the 100 attempts (52%), the dentists failed to correctly identify the patient's gender. They were successful in accurately identifying the gender of the remaining 48 (48%). The exact two-tailed significance value of 0.764 indicates that the observed proportions of pictures where gender was identified correctly (48%) or incorrectly (52%) did not differ significantly from an expected 50-50 split. 

**Table 5:** Dentists' performance in identifying patients’ gender: First Attempt A p-value of >0.05 is considered non-significant. N/A: not applicable.

	Category	N	Observed Prop.	Test Prop.	p-Value
Were the dentists able to identify gender of the patient the 1st time	No	52	0.52	0.50	0.764
	Yes	48	0.48	N/A	N/A

In other words, the dentists' performance in identifying gender from these pictures during the first attempt was no better or worse than random chance. The non-significant p-value suggests that the dentists encountered challenges in this task, as their accuracy rates did not exceed what would be expected by chance alone. 

Table 5 presents the results of a statistical test examining whether dentists were able to accurately identify the gender of patients from a set of 10 pictures during the first attempt. For 52 out of the 100 attempts (52%), the dentists failed to correctly identify the patient's gender. They were successful in accurately identifying the gender for the remaining 48 (48%). The exact two-tailed significance value of 0.764 indicates that the observed proportions of pictures where gender was identified correctly (48%) or incorrectly (52%) did not differ significantly from an expected 50-50 split. 

In other words, the dentists' performance in identifying gender from these pictures during the first attempt was no better or worse than random chance. The non-significant p-value suggests that the dentists encountered challenges in this task, as their accuracy rates did not exceed what would be expected by chance alone (Table 5). 

Table 6 shows the cross-tabulation of the dentists’ responses at the two time points. During the first attempt, out of the 53 inputs where 10 dentists failed to identify the patient's gender correctly, 27 answers (50.9%) involved the same pictures, and they were unable to identify the gender in the second attempt as well. On the other hand, out of the 47 answers where dentists successfully identified the gender in the first attempt, 25 answers (53.2%) were pictures where they could not identify the gender correctly in the second attempt.

**Table 6:** McNemar test results: dentists' performance in identifying patients’ gender across two time points A p-value of >0.05 is considered non-significant.

		Were the dentists able to identify gender of the patient		p-Value (McNemar Test)
		No	Yes	
		N(N%)	N(N%)	
Were the dentists able to identify gender of the patient the 1st time	No	27 (50.9%)	25 (53.2%)	1.000
	Yes	26 (49.1%)	22 (46.8%)	

Conversely, out of the 47 inputs where the 10 dentists identified the gender correctly in the second attempt, 22 answers (46.8%) were pictures where they had failed to do so in the first attempt. Similarly, among the 53 inputs where dentists could not identify the gender in the second attempt, 26 inputs (49.1%) involved pictures where they had successfully identified the gender priory.

**Figure 3:**
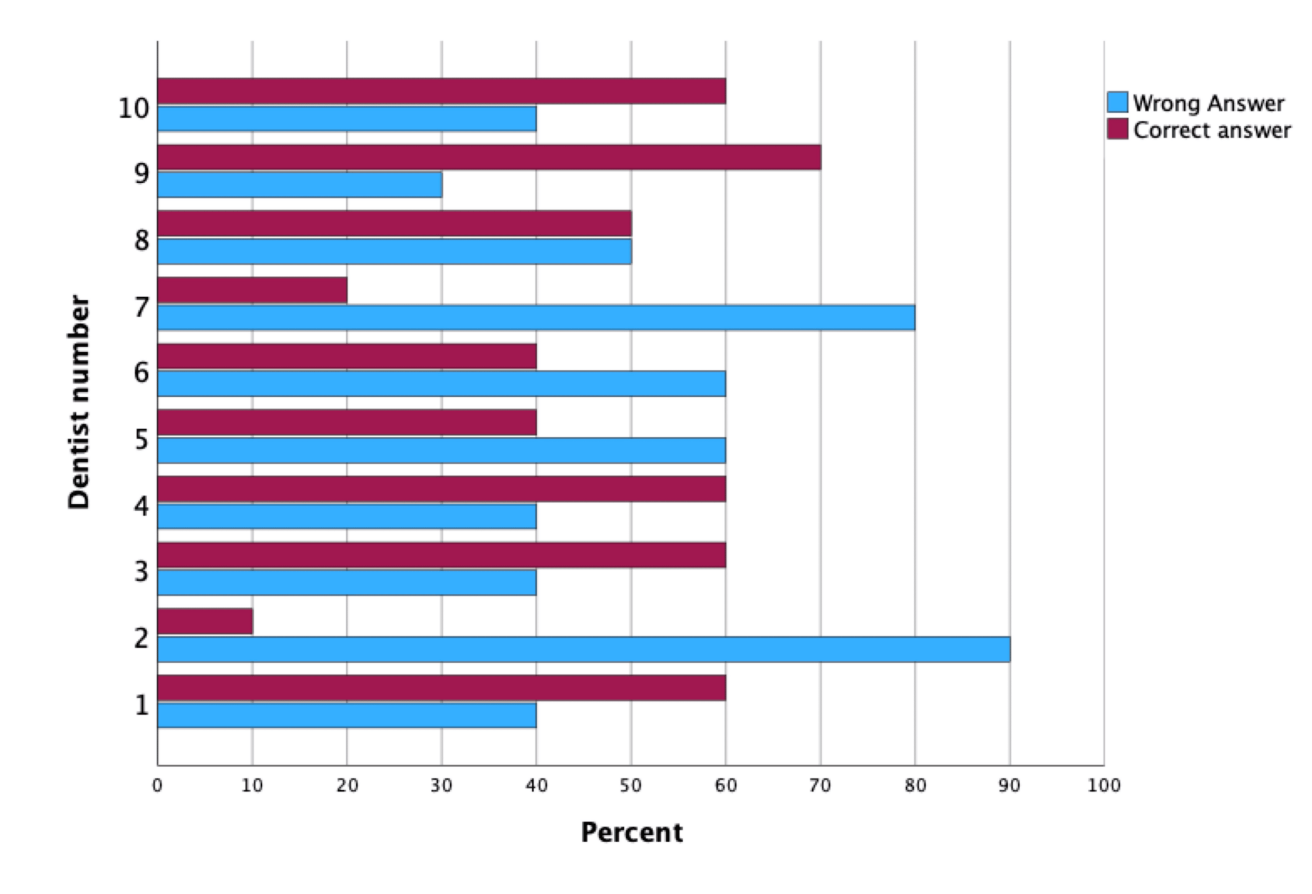
Clustered bar chart for frequency of correct and incorrect answers for each dentist in identifying patient’s gender at second time point

**Figure 4:**
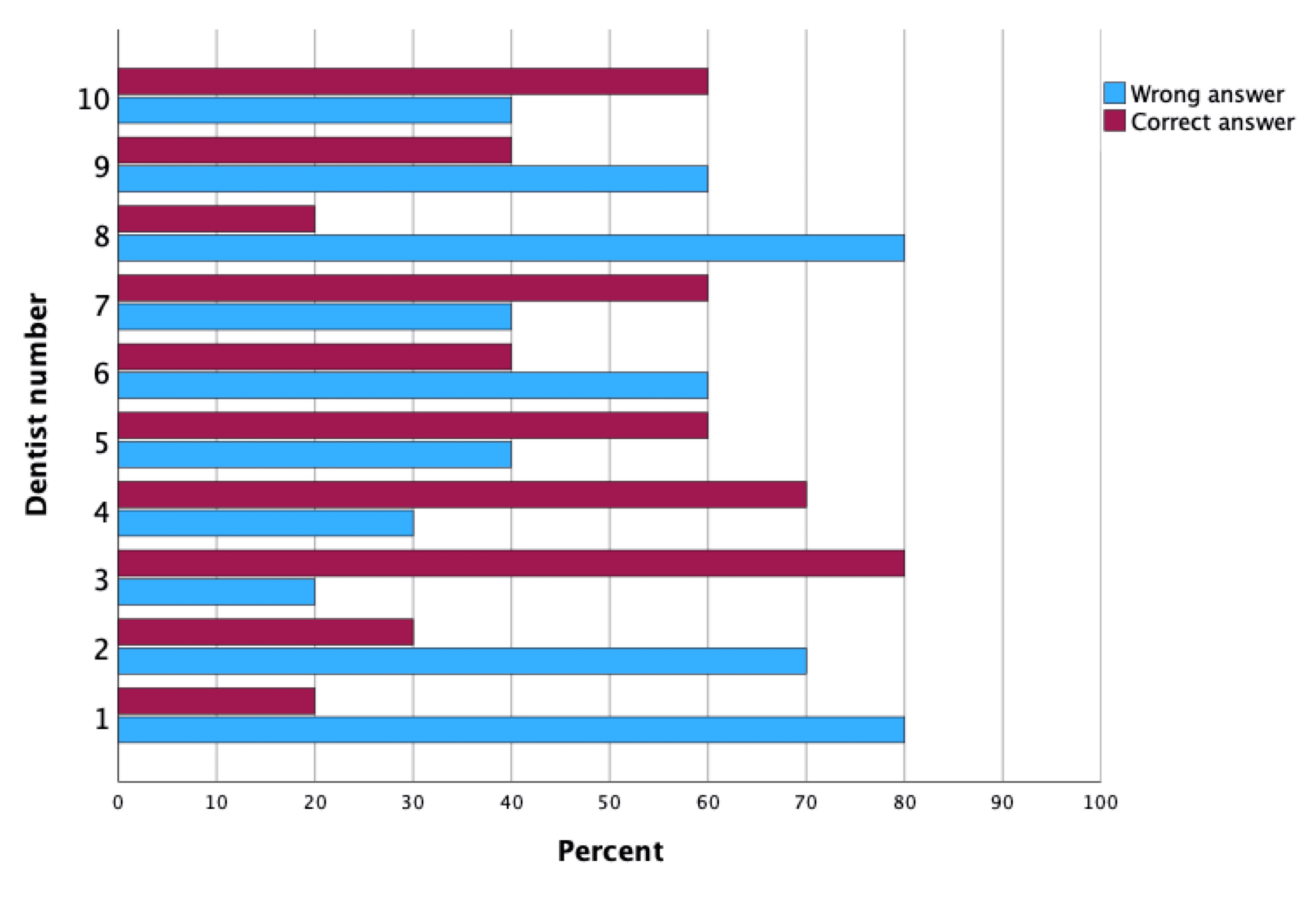
Clustered bar chart for frequency of correct and incorrect answers for each dentist in identifying patient’s gender at first time point

The non-significant p-value suggests that the dentists' performance in identifying the patient's gender from pictures did not differ significantly between the first and second attempts. Their accuracy rates remained consistent across the two sets of pictures. Figures 3, 4 are clustered bar charts highlighting the difference in the percentage of correct and incorrect answers by each of the 10 dentists participating twice in this study (Table 6, Figures 3, 4).

## Discussion

In previous studies, various aspects have been investigated to establish correlations between tooth shapes, genders, and facial structures [13]. However, routinely correlating these factors with the patient’s gender is considered difficult [9].

It is important for dental professionals to be familiar with the tooth shape, size, and color, especially for restorative treatment, to keep the teeth in synchrony with the face and possibly gender. Our raised question of whether dentists can identify the gender based on visualizing anterior teeth only to see if sexual differences may be recognized. We expected dentists to focus on traits such as tooth shape, including lines and angles, size, and color. Also, by looking at the gingival margin, gingiva, and the smile as a whole try identifying the gender. In our study, tooth morphology was linked to gender significantly by the participants, which showed that despite some differences, it may still be identified. This may be deduced by the familiarization and high exposure of dentists to patients, which gives them the upper hand in determining gender by looking at the teeth.

Orthodontists did not perform significantly better than other specialties, which may be due to the fact that orthodontists concentrate on the occlusion and position of teeth rather than shape and morphology. Prosthodontists and dentists performing restorative treatments may have better expertise in deciding the patient’s gender and acquaintance when matching anterior restorations to fit patients’ face, body, and gender.

As aforementioned, women are more likely to be perceived to have smaller, rounded, tapered teeth, which may be the reason behind Figure 1 (shown above) being the most photographed correctly as female. Whereas, in Figure 2 (shown above), this male patient also has small and rounded teeth, which may be the reason why most participants labeled him incorrectly as a female. This coincides with another study done by Mahn et al. in 2018 that showed no correspondence exists between tooth shapes and patient genders [12]. However, there are some studies that demonstrate that sex determination using dental features primarily relies on comparing tooth dimensions between males and females. In modern human populations, males tend to have larger tooth crowns than females, which may be attributed to a longer duration of amelogenesis for both primary and permanent dentitions in males [14].

Clinical implications

This area of research has the potential to bridge forensic, clinical, and anthropological disciplines, providing a multifaceted tool for both medical practice and scientific inquiry. Also, esthetic features may be shown or highlighted specific to the patient's gender while also asking the patient about their preferences. In addition to that, gender-specific features may be explained to the patients, for example, when finishing a restoration or enameloplasty of the lateral incisor, that it is more rounded for females. This would help the patient see why the restoration and their smile are customized to them.

Limitations

A limitation in this part of the study was that two cases had non-carious tooth loss or attrition, and so the tooth morphology was affected, which may affect the results. It cannot be estimated how much attrition affected the tooth shape or the original tooth shape before the occlusal wear. This is due to the teeth belonging to parts of the body that show continuous transition of features [15].

The photographs provided show the teeth in habitual occlusion, or occlusion where maxillary and mandibular teeth fit in maximum intercuspation. In our study, all the photographs were post-orthodontic treatment, and so their bite of convenience would be the maxillary teeth overlapping the mandibular teeth with an overbite of approximately 3 mm. Therefore, the mandibular teeth are not visible for sexual determination, as with the maxillary teeth in our photographs. Also, this evaluation is different from forensic sexual evaluation, as with forensics, teeth may be visualized from all aspects. Studies show that studying morphological differences of the teeth between males and females can be applied to identify the gender, especially in forensic dentistry [16]. 

Moreover, a ‘cannot be determined’ answer would filter out indeed correct answers. The second participation or analysis was done by only 10 participants two months after their first contribution again showing that most of their answers may be speculated when determining the gender with no clear reasoning.

## Conclusions

Overall, the study's findings suggest that both orthodontists and dentists from other specialties could identify an individual's gender based on photographs of tooth morphology with statistical significance, although the level of significance varied between the groups. However, there was no significant difference in the performance of orthodontists compared to dentists from other specialties in this particular task. Furthermore, our study showed that dentists are able to identify or determine gender based on photographs, which is inconsistent with our starting hypothesis. The findings revealed that both orthodontists and dentists from other specialties performed similarly, with no significant difference in their abilities.

## Appendices

Title of the project: Gender Determination Through Dental Morphology- A Cross-sectional Study.

The aim of the study is to assess if dentists are able to Identify the gender of the patient by evaluating the dental morphology using intraoral photographs post-orthodontic treatment.

The procedures involved in this study include: Answering 10 questions on this questionnaire using the photographs provided, which should only take you about 5 minutes to be completed.

There are no risks to you if you participate in this research. Your participation will increase knowledge about this important issue. All information collected will remain confidential. Neither your name nor address will be recorded in any assessment. There is no obligation or compulsion for you to participate, and you have the freedom to agree or not agree to participate. This will not have any effect on you. You may withdraw from the research at any time. This research does not include any medical experiments or taking biological samples.

Please indicate (✔️) below if you wish to participate or decline to do so:

I wish to participate

I do not wish to participate

Thank you for your cooperation…

Investigators’ names: Dr. Samaa Alsaraf and Dr. Hashem Alsaegh

Supervised by: Dr. Mohammad Qali

Date: __________________

Sample No.__________________

Participant’s dental speciality: 

General Practitioner Orthodontist Periodontist 

Prosthodontist Endodontist Advanced General Dentist 

Oral Surgeon Pedodontist Others:___________________

**Table 7:** Based on the following intraoral photographs taken post-orthodontic treatment 1-10 provided, tick (✔️) the appropriate box:

Case	Do you think this patient is male or female, depending on the dental morphology?	
	Male (M)	Female (F)
1	M	F
2	M	F
3	M	F
4	M	F
5	M	F
6	M	F
7	M	F
8	M	F
9	M	F
10	M	F
